# Ten simple rules for navigating the computational aspect of an interdisciplinary PhD

**DOI:** 10.1371/journal.pcbi.1008554

**Published:** 2021-02-18

**Authors:** Sabrina Islam, Christine A. Wells

**Affiliations:** Centre for Stem Cell Systems, University of Melbourne, Melbourne, Australia; Carnegie Mellon University, UNITED STATES

## Introduction

A PhD project in many ways is akin to an adventure quest, and like any quest, you may quickly find yourself in uncharted territories. The interdisciplinary nature of modern research brings new treasures to the adventurer—the internal rewards come in forms of mastering skills and overcoming personal boundaries; and the external rewards come in the forms of discovery, intellectual discourse, and travel. For many of our colleagues, the sudden arrest of a lab-based adventure because of the Coronavirus Disease 2019 (COVID-19) pandemic measures has forced an unexpected redirection of their research activities towards data analysis. The first author had the opportunity of undertaking such a quest into computational phylogenetics for her first thesis chapter. As she was equipped with a background in molecular biology and genetics but not computation, this first chapter served to her as a launching pad into the previously uncharted sea of data science.

**Fig 1:**
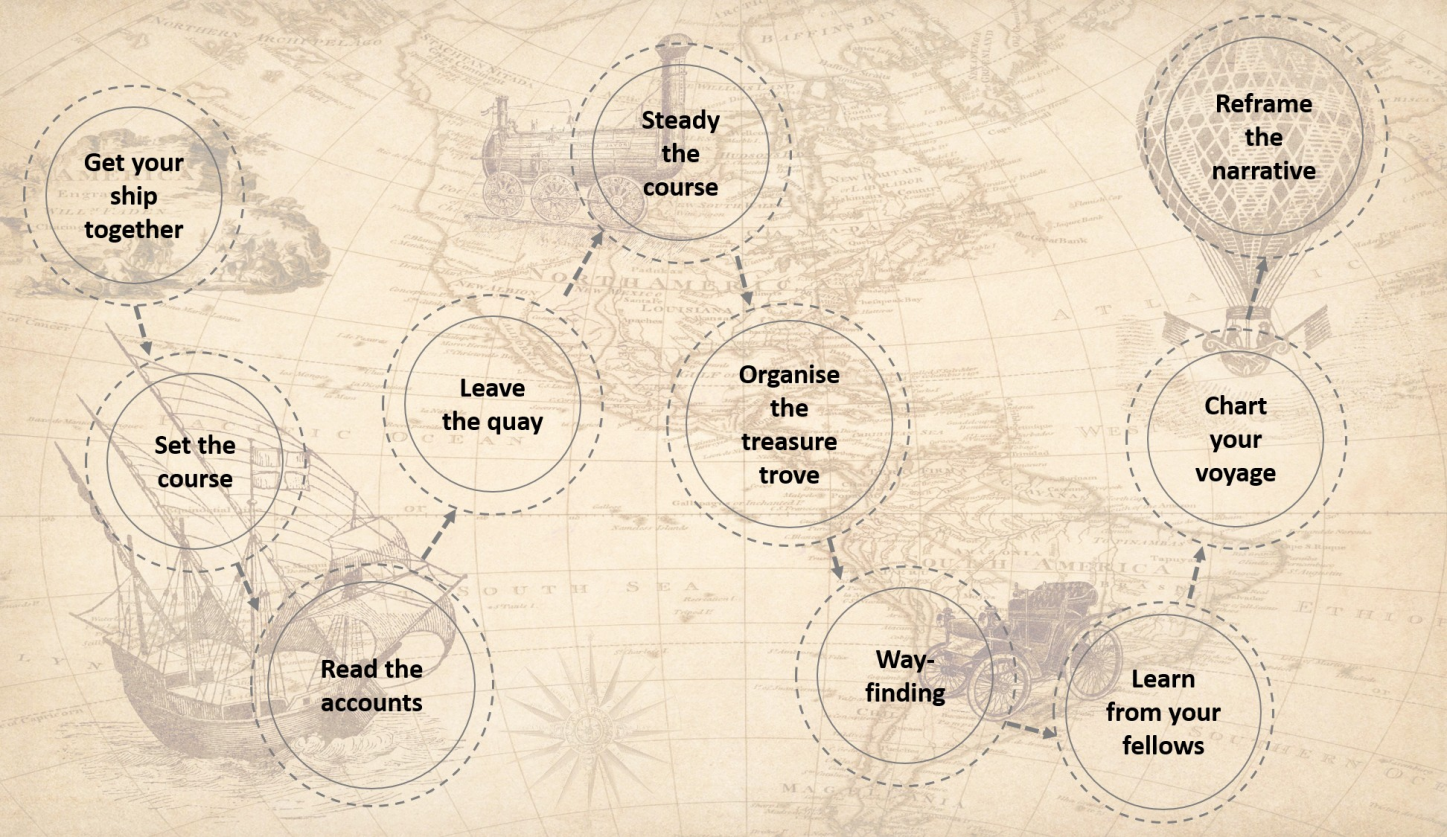
Ten simple rules for launching a research career in computational biology. The backdrop image edited from an illustration of @Dorothe. *Image credit*: https://pxhere.com/en/photo/1442539.

As with any new adventure, fear of the unknown can put unnecessary monsters onto the map of your PhD, but these can be defeated with a little advanced planning. The authors hope the following tips will benefit several audiences. While written from the perspective of the wet lab biologist transitioning to computational biology, we anticipate these rules will be useful to interdisciplinary researchers mastering a new skill set, or transitioning to a new research topic, or, like the first author, switching their specialisation. Any research quest benefits from bilingual adventurers, versed in both wet lab and computational biology. These tips come from a data science rookie, who switched her research career from wet to dry lab shortly before the pandemic lockdown, and a data science veteran with 16 years of experience and mentorship reflections in computational stem cell biology. Here, we outline our journey in a quest format for the future interdisciplinary explorers (Fig 1). This write-up is, of course, more philosophical rather than an actual step-by-step walk-through. To paraphrase Captain Barbossa from the Pirates of the Caribbean, they are more of guidelines rather than an actual set of rules.

### Rule 1: Get your ship together: Structure the fundamentals of your workspace

Transitioning into a space such as computational biology requires you to learn the vocabulary and the grammar of several aspects to carry you through your PhD. The first aspect encapsulates the biological concepts of the field that you are journeying into, for which a background in biology will greatly facilitate understanding of prior ideas as well as knowledge gaps. This is the hull of your ship, ready to be outfitted with the knowledge gained through your journey. The second is technical proficiency, i.e., mastering 1 (or more) coding language(s) or software environments—this is the engine room of your ship. The third aspect needed to outfit your voyage is familiarity with the data science concepts relevant to you. This is your compass. The field of statistics is a big one, and you will need to navigate key assumptions and potential pitfalls that come with your data structure and the statistical models that you chose to apply.

Defining your program with such broad topics may be challenging initially, so it is necessary to structure the self-learning process. The usefulness of a resource depends largely on your personal aptitudes. Some may benefit from chapters or excerpts from books such as *Evolutionary Genomics* [1] or *Introduction to Data Sciences* [2]. Some may absorb skills from actionable steps from massive open online courses (MOOCs) such as Coursera (https://www.coursera.org/), Udemy (https://www.udemy.com/) and such, or a quick primer from DataCamp (https://www.datacamp.com/) or Software Carpentry (https://software-carpentry.org/). Hands-on workshops on software or coding languages from your institution or elsewhere can provide opportunities not only for immersive learning [3] but also for collaborative learning [4]. Of course, in the beginning, you may feel intense fear of missing out (FOMO) regarding every workshop in your vicinity. Learn to prioritise and budget your time, and discuss with your advisors the utility, practicality, and congruity of the workshops with your research.

Familiarise yourself with the foundations of data file formats. One part of this is to understand how to reformat your data to be compatible with your software of choice. This likely involves importing text files from databases, reading it into the coding environment, and subsetting it to fit your experimental design. Another key task is learning the identifiers of data in your field, such that you can find relevant information between databases, and map your own findings to these meaningfully. You may find gratification in producing and customising beautiful plots from packages such as ggplot [5] or plotly [6]. These are generalisable skills that give you agility through your journey and will serve you well on future voyages. More tips accompanied with beautiful infographics can be found on “Ten simple rules for biologists learning to program” [7].

### Rule 2: Set the course of your research

It can feel overwhelming to start your own research journey, when you lack experience, and in an area where expertise is gained over the course of your studies. The veteran adventurer knows that even well-trodden paths can turn up new treasures if a fresh perspective is applied. With technology advancing the resolution of the lens with which we study biology, your quest may reveal a deeper understanding of your chosen system simply because you can navigate the sea of data with a biological lens, or vice versa.

Nevertheless, you will need to frame the course of your studies by identifying a knowledge gap that you can uniquely address. The post-omics world offers new entry points and perspectives into biological questions that were previously unnavigable, but that also means that there are risks for the unprepared. To avoid spiralling into a philosophical maelstrom, define the premise of your study and its boundaries early on. Avoid overfocus on the methodology by charting how the steps will advance your overarching research goal. Equally important as locating a question is determining your experimental design. This will encompass 2 aspects: (1) locating the right methods; and (2) locating the benchmarks to determine whether your findings are sufficient. Expect those research aims to evolve—their scopes may broaden or narrow as you journey further. This is part of the joy of the voyage of discovery.

Your exploration should ideally include heuristic-driven exploration, one that fuels understanding of the data, its structure, and the methods that you apply. Alongside the heuristic-driven exploration, we also encourage you to add a hypothesis-driven perspective. Too often, “omics experiments are considered to be ‘hypothesis-free.’” We believe that analysing data with a set of testable hypotheses in mind helps to answer biological questions that a plain descriptive approach may overlook. One advantage of coming from a different discipline is that your question is likely to be grounded in the experience and perspective that you bring with you. Embrace your differences. Your former training in “classical” biology may even allow you to switch to the hypothesis-driven dimension more naturally, as you would be keener on fishing the biology from a sea of data. Certainly, the question that you wish to ask should inform the test that you apply, rather than the other way around.

### Rule 3: Read the accounts of those who voyaged before: Read the literature critically

Rule 3 ought to be exercised in parallel with Rule 2. Structuring your reading will help you avoid the frustrations that accompany a slow and unfocused journey. Your library catalogue should include the watershed papers on the field, even though they may not be directly relevant to data science. As you start to map out a research narrative, you will also find yourself reading papers that document computational methods, computational tools, and database reports. These are the papers that will solidify your skills and help you orient your course. The information they supply will underline how a computational approach could add to the field that you are studying. There are several strategies to practise purposeful readership. Drafting a literature review will help to organise the experience. Presenting your newly absorbed knowledge to an audience (supervisors, fellow students, or lab group) is another option to organise your research thoughts. You could also consider anchoring a summary of each paper to a position in your thesis.

You may find that science does not always reflect objective, and constant conclusions, but can be subjective and provisional. Previous voyages may have been shaped by the constraints of the day—for example, small sample size, inaccessible resources, or assumptions that should be reevaluated in the light of new knowledge [8]. Citation propagation can lead you into shallow water, especially if these depend on obsolete methods or draft genomes [9]. Learn to question and challenge the assumptions and approaches that authors have employed as you read. Do the assumptions of the study stand up to recent progress in the field? Is there sufficient detail to reproduce the study findings? Of course, you will quickly realise that “reproducibility” is also predicated by context, impacted particularly by genome references or software versions. Correspond with an authorship team if you cannot recreate their results—dialogue helps build an understanding of their approach, proficiency in the field, and importantly, may allow for any discrepancies in the data to be corrected in the public record. Understanding how and where data is reused, particularly when plots from the same data vary drastically among publications, is a useful way to reflect on individual methodology. “Ten simple rules for developing good reading habits during graduate school and beyond” offers great tips on healthy and intelligent reading habits [10].

### Rule 4: Don’t let procrastination anchor you to the quay: Overcome initiation anxiety

The most anxiety-inducing act for the first author was to start to code and build phylogenetic trees. Fear of the unknown resulted in research doldrums that was best solved by dipping her oar into the data. Partnering reading with coding allowed this novice to gradually move from theory to application. As this is a period with a steep learning curve in your research topic and your research methodology, remember to give yourself the time you need to review your ideas and your approach. Remind yourself that the dry lab is more forgiving and less expensive than the experiment that created the data in the first place—you can afford to test the waters a few different ways.

At least initially, we advise you to choose a widely used coding language—the documentation, the library of tools, and the community will support you. First, practise with small, toy datasets (which can be supplied from the workshops or online tutorials or even accompany an exemplary paper in the field), build expertise before expanding into larger datasets or more complex workflows, and then channel your data. You can also learn interactively through packages such as swirl (https://swirlstats.com/students.html). The resources we found useful are summarised in Table 1. Launching your study by reproducing a previous study at a smaller scale is helpful to review study design as well as posit the literature in your topic. For example, we chose to shorten a published vertebrate tree with a quick tree—with only primate sequence and minimal computations to check if the branching orders of the two agreed.

**Table 1:** A list of resources that we found useful while learning and mentoring how to code.

Topic	Resource	Link
Quick R primers	R Tutorial’s free R introductory course	http://www.r-tutor.com/r-introduction
	DataCamp’s free R introductory course	https://www.datacamp.com/courses/free-introduction-to-r https://www.statmethods.net/
	R set-up primer from a PhD student	http://web.cs.ucla.edu/~gulzar/rstudio/basic-tutorial.html
Exhaustive R education	Introduction to Data Science by Rafael A. Irizarry	https://rafalab.github.io/dsbook/index.html
	R for Data Science by Garrett Grolemund and Hadley Wickham	https://r4ds.had.co.nz/
	Cookbook for R	http://www.cookbook-r.com/
Video teaching and learning	Learning R with videos	http://jeromyanglim.blogspot.com/2010/05/videos-on-data-analysis-with-r.html
Interactive learning	Learning R with swirl	https://swirlstats.com/students.html
Data literacy platforms	Software Carpentry	https://software-carpentry.org/lessons/index.html
	DataCamp	https://www.datacamp.com/
	Code academy	https://www.codecademy.com/
	Code school	https://www.pluralsight.com/codeschool
	Rosalind	http://rosalind.info/problems/locations/
MOOC platforms	Coursera	https://www.coursera.org/
	Udemy	https://www.udemy.com/
	EdX	https://courses.edx.org/
Problem-solving platforms	StackExchange	https://stackexchange.com/
	StackOverflow	https://stackoverflow.com/
	Biostars	https://www.biostars.org/
	Seqanswers	http://seqanswers.com
	ResearchGate	https://www.researchgate.net/
	Troubleshooting R codes	https://rseek.org https://www.r-project.org/help.html https://community.rstudio.com/
	R package documentation and publications	https://cran.r-project.org/ https://www.bioconductor.org/
	Google	https://www.google.com/

The list reflects our personal experiences, with a focus on mastering R and the coding skill set.

MOOC, massive open online course.

Similarly, taking time to scope methodological articles helps you to locate and understand the theoretical basis of the tools, databases, and packages (Rule 3) that you will draw on in your studies. While clicking around is not advised anymore and most experts recommend letting go of spreadsheets and graphical user interface (GUI) altogether, the first author found it useful to make the transition from clicking to coding gradual. In her early attempts to learn phylogenetic tree building, she found it reassuring to familiarise herself with parameters and visualisation options by creating a small tree output using the click-format of MEGA-X [11] before building a BEAST2 [12] tree with command line in the cluster.

### Rule 5: Adjust the sail to steady your course: Managing your project with patience

Realise that everything will be very slow, at least in the beginning. Learning new skills will take a ridiculous amount of time, so will analysing, interpreting, and debugging codes. Understand that while you can do anything, you can’t do everything, so you will need to calibrate your initial research goals quite a bit. Your ability to undertake this journey is not synonymous with your capacity to perform every task. The latter skill set will develop only through perseverance throughout your studies, as you will gain soft skills that will optimise this sailing experience and many more to come. As frustrating being in the rookie seat while having high expectations can be, remember to practise compassion and patience with yourself.

**Fig 2:**
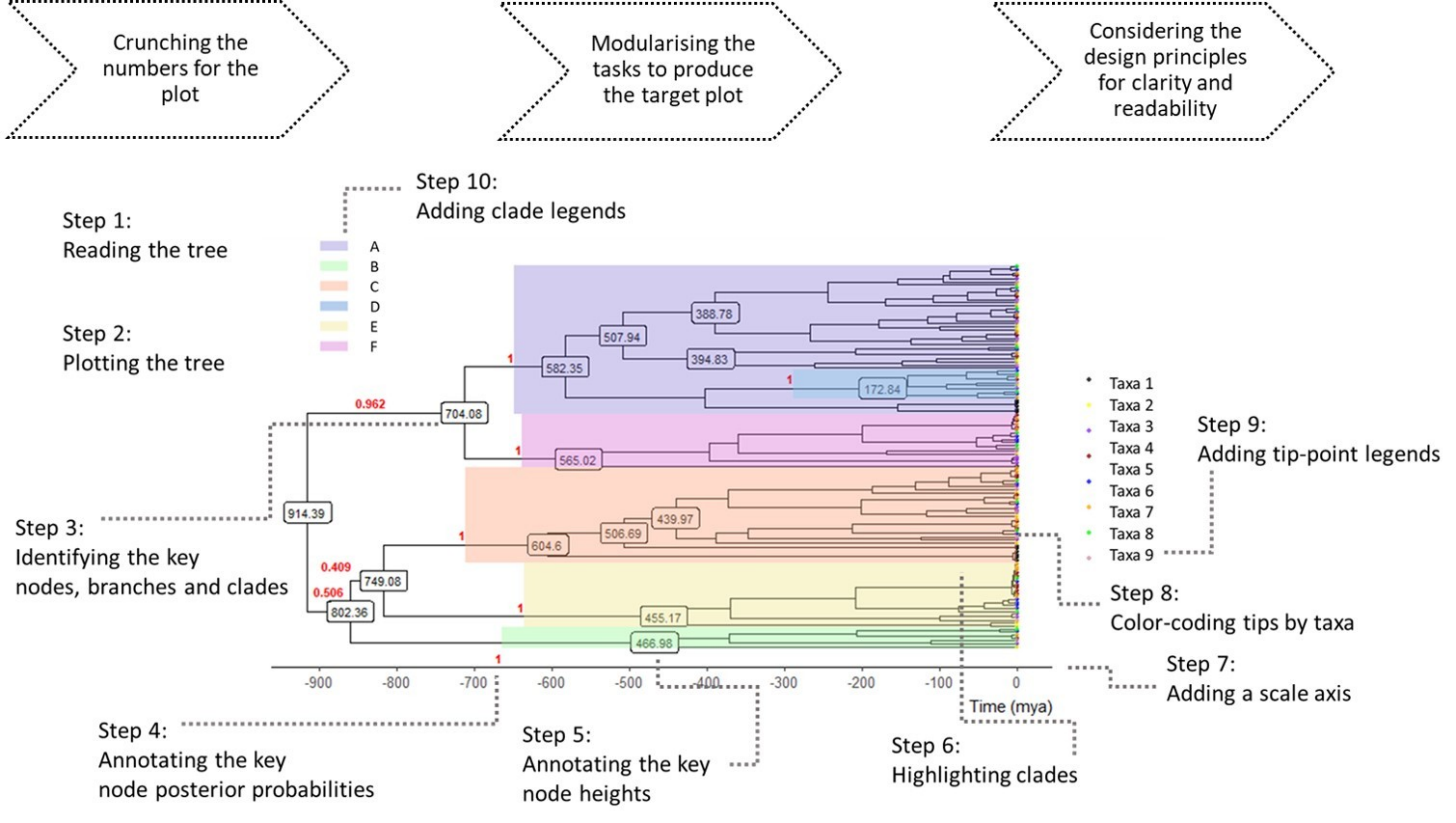
Modularisation as a project management strategy. Here, a time tree visualisation is broken down to 10 modular steps.

Training in data science can be both intellectually and emotionally challenging. Productivity in coding can be very hard to measure, and the learning curve is not always linear, which can make the loss of motivation rather easy. To make the task more palatable, break the entire project to smaller goals, and break those goals down to bite-sized modules. Tackle those modules one at a time. For example, annotating a large tree can be broken down to reading the tree data; plotting the tree; identifying the important nodes, branches, and clades; annotating those nodes, branches, and labels; labelling the tips meaningfully; placing scale bars or scale axis; and finally, formatting the tree for publication (Fig 2).

To make the most of incubation periods, plan well. Your planning system depends very much on your style—some people prefer checklists (https://gettingthingsdone.com/what-is-gtd/), some timeboxed calendars (https://hbr.org/2018/12/how-timeboxing-works-and-why-it-will-make-you-more-productive), some rapid logging (https://bulletjournal.com/pages/about). But while planning styles vary, anyone can benefit from some form of planning. Slot number-crunching or sample building in between code runs. Finally, punctuate your analysis with writing sessions that will later serve as material for your thesis.

Think about coding efficiency as part of your laboratory tasks. Compile and test-drive a script with a trimmed dataset. Doing so would let you fish for bugs as well as to outline the stages of analysis. Learn to automate what you can. Leverage existing tools to move forward rather than reinventing the wheel. Your critical readership from Rule 3 will be useful here as you will be informed of the existing tools where you can feed your data. Anything that is repeated more than thrice should become a script or function [13]. Definitely delegate massive sampling and computation to cluster computing. Copy-paste intelligently—the scripts provided by your institution’s high-performance computing (HPC) cluster may or may not match the command line from the software documentation. Smart googling can dramatically speed things up. But to do that, learn the anatomy of the error message and the language of the computer community, which may take months of practice.

### Rule 6: Have your treasure trove organised: Manage your data

While practising responsible data husbandry is highly encouraged in any discipline, being organised is imperative in computational biology as you deal with massive amounts of data, codes, and files. FAIR data principles of Findable, Accessible, Interoperable, and Reusable are the agreed cornerstones of open data science, and these principles hold value on the personal level as much as they do at an institutional level [14]. Organising your data, methods, and results may seem like “busy work” when your time and thinking is already stretched, but academic housekeeping and data hygiene are what differentiate between good and bad science. Being organised and intentional will not only make your own research easier for you to reproduce, reuse, and recycle; it will let you communicate and collaborate with transparency.

Before commencing data collection, invest in a proper file management system and craft a data management plan (DMP) if needed. As you format and organise data, the Unix filesystem and shell scripting would come in handy. Treat your future self with kindness and incorporate a readme or standard operating procedure (SOP) file in every project folder and subfolder. Thoroughly document and annotate every stage of your data analysis. Record your methodology, and maintain a research journal/lab notebook—a digital note-taking software such as Evernote (https://evernote.com/) or OneNote (https://www.microsoft.com/en-au/microsoft-365/onenote/digital-note-taking-app) are much better suited for this purpose than a paper notebook—we recommend OneNote ([15] is an excellent review of the established note-taking apps). Alternatively, electronic ecosystems such as Jupyter Notebook [16] or R Notebook [17] allows seamless knitting of analysis and result and facilitate reproducibility. We encourage you to elaborate on your thinking as you annotate your code—keeping the “doing” linked to the rationale of the experiment. Write down every research decision and their thought process. Which samples did you discard or select? How did you accommodate biological assumptions and biases? How much sampling was needed to produce reliable parameters? What was your measure of statistical support?

Be ruthless in curating the data that you include in your studies. Large projects such as Stemformatics.org discard as much as 30% of the public data that they evaluate [18]. If the experimental design or quality of the data is not fit for purpose, then it will confound rather than assist your journey. Data curation also requires good management of your data resources throughout your project. We advise you to educate yourself with the data storage and curation that you are entitled to by the nature of your data, the policy of your institution, and your research funders. Reference [19] offers some practical advice in this arena. Try to master some form of version control—git/GitHub, Subversion, Bazaar, Mercurial, and Bitbucket are all good options to choose from. Specific to coding, make your codes readable and your data tidy [20,21]. Comment generously on every chunk of code—this would make backtracking and debugging much less painful. Document and catalogue old codes for recycling when needed. Two excellent articles on documenting and sharing codes are “Ten Simple Rules for a Computational Biologist’s Laboratory Notebook” [22] and “Ten simple rules for writing and sharing computational analyses in Jupyter Notebooks” [23].

Finally, store the precious results of your analysis with care. Treat your output figures and tables as entities that are separate from the data and can be reproduced by running the same code. Annotate the “raw” and the edited versions of images properly. Name each output file meaningfully. Consider your final product in terms of readability (Are your axes labelled? Is the colour scheme part of your narrative? Is the figure of high enough resolution to go into a paper/thesis chapter?), and see Rule 9 for more on this topic.

A noninclusive list of resources to manage and communicate (discussed shortly) the computational biology project is summarised in Table 2.

**Table 2:** A list of useful resources to organise computational biology research.

Topic	Resource	Link
Script documentation	R Markdown	https://github.com/rstudio/rmarkdown
	Jupyter Notebook	https://jupyter.org/
Digital note-taking	Evernote	https://evernote.com/
	OneNote	https://products.office.com/en-au/onenote/digital-note-taking-app
Time management strategies	Timeboxing	https://hbr.org/2018/12/how-timeboxing-works-and-why-it-will-make-you-more-productive
	Checklist/GTD (getting things done)	https://gettingthingsdone.com/what-is-gtd/
	Rapid logging	https://bulletjournal.com/pages/about
	Kanban method	https://blog.trello.com/kanban-101
Research output communication	The grammar of graphics	https://cfss.uchicago.edu/notes/grammar-of-graphics/
	Writing in the Sciences	https://www.coursera.org/learn/sciwrite

The list summarises the optimising, managing, and communicating strategies we benefited from.

### Rule 7: Way-finding: Reflect on progress and review course

Formidable statistician George Box famously said, “All models are wrong, but some are useful” [24]. When modern physics emerged alongside classical physics, it introduced a lot of uncertainty and relativity in how we model the universe, but those models were useful anyways. Computational biology is similar in that sense—computational models may not represent the objective truth, but they certainly aid our subjective understanding of biological systems. Treat your results as inferences, not absolute truth. Think about ways to reflect on, and report the credibility of your outputs. Address the limitations of your study as you go, and think about how to refine it.

Not only do you need the time to do, but you also need the time to think. Take the time to adjust to the new headspace of the discipline. Reflect on what you did, and review what worked versus what did not, and why, and actively transform your methods based on that. Using someone else’s pipeline blindly is like treading new waters with someone else’s map. Instead, tailor your workflow to fit your quest—the questions at hand. Carefully consider data source, sample size, experimental design, and your computational questions. These “thought processes,” although time-consuming, are valuable learning opportunities. As Maui said to Moana in the iconic Disney movie, you need to know where you have been to know where you are headed.

Progressing through reflections should be a process shared among the stakeholders of your research. Understand that your supervisors and committee members are not as intimate with your research as you are, so update them periodically of your journey. Make the most of your meetings with pre-meeting agendas and post-meeting notes, more so when you start fresh in a new area. Connect with your lab members and committee members, be it in “meat space” or virtual space. The authors differ in their preferred approaches: One feels much safer brainstorming virtually over e-mails or google docs, whereas the other enjoys a face-to-face discussion, perhaps armed with a whiteboard to map out ideas—so use whatever mode of communication works for you and your team. To make the best of you and your advisors’ time, articulate the challenges you are facing to them efficiently, clearly, and transparently. Stakeholders in our research extend beyond our committee or lab—the pharmacologists who will build upon our finding to engineer drugs, the healthcare professionals who will put our discovery to practice, the peers and students who will add increments to our knowledge, and the community that are funding our research (directly or indirectly) and whom we wish to benefit—are all part of our research narratives. Finding ways to engage with these different audiences will also serve as points of reflections and connect our data to real-world, tangible meaning.

### Rule 8: Learn from your fellow excursionists: Find the communities who will enrich your studies

Good science relies heavily on being able to draw on and contributing to the collective knowledge of experts. Equally important as locating a topic is finding the community of experts to contribute to. The role of conferences is obvious in this regard, but we encourage you to look towards additional informal assemblies of peers and mentors to enliven your journey. Finding people who will encourage and challenge you is essential. In our experience, good mentorship is less about actual hand-holding into coding and more about developing a discerning eye for quality data analysis and critical thinking. Non-coder peers can be especially valuable in testing the soundness of your ideas, so value their “outsider’s perspectives.” We found that one of the best ways to transition into a new field was to shadow an experienced player. The first author had the privilege of learning from the developers of the BEAST2 themselves by simply shooting them an e-mail. Depending on your learning style, you may also benefit from coding communities within your lab, university, or city, such as the R-Ladies (https://rladies.org/) or hackathons. These groups often draw on members with different backgrounds, from industry, academia, a savvy research assistant, or an undergrad with more background in coding than you. Being an active participant in these communities is key: Organisers of workshops may be happy to answer your questions, but equally may be keen for you to contribute your own expertise to the next session. Virtual networks are also excellent places to meet like-minded scholars. Google, StackExchange, and StackOverflow are your best friends from now on. Online learning communities as such as Coursera have discussion boards for learners. You can further subscribe to mailing lists of software or language user groups. Feel free to copy and run codes from online forums, documentation (in CRAN (https://cran.r-project.org/) or Bioconductor (https://www.bioconductor.org/install/)), publications, or a lab member, but remember to give appropriate credit and check license while doing so. An excellent resource for accessing online scientific communities is [25].

Finally, consider your work in the context of the teams around you. Sharing your research ambitions, and contributing to others, is one of the best parts of your laboratory life. Understand that outsourcing some of your work to someone more suited does you no discredit. If anything, this would also serve as a learning and enriching experience for you in project management. Shared approaches expose you to alternate ways to approach a complex problem. Likewise, being willing to work on other projects within the lab broadens your technical horizons and builds collaborations that may extend beyond the scope of your own thesis, but will no doubt enrich your journey.

### Rule 9: Chart your thesis cartography: Share the findings of your voyage

As you move closer to the finishing line of your analysis, you will start thinking about communicating your research. It could be a thesis chapter, or a paper, or a poster, or discussed as part of your final talk. Whatever the format, the delivery will matter just as much as the content. Don’t assume that your audience will have seen the output of your analysis before—take the time to maximise the clarity and brevity in your tables and figures. Would your past, molecular biologist self be able to interpret the figures that your current or future self is producing?

Start by combining the legends and captions, such that each table and figure should be a stand-alone entity. Be sure that the visualisation mode that you choose is appropriate to the data. For example, do not plot numeric variables such as alignment score or sequence distance against categorical variables such as taxa name. Use colour as part of your narrative by colour-coding motifs, alignments, and clusters coherently, meaningfully, and readably. Expect that if you are producing a visual output, your audience will expect to be able to read the detail, so don’t cram labels in badly aligned, tiny fonts. Prefer human-oriented information to computer-oriented information. For example, database accession numbers or coded sample IDs can be substituted with plain language sample descriptions or gene symbols where relevant. If the labels overcrowd, use acronyms or colour blocks. Strip the figures and tables of any visual clutter—annotate only the relevant data points and tabulate only the key numbers. Remember to include the scale as well as the measures of the axes. Packages such as ggplot [5], plotly [6], and matplotlib [26] offer great quality and customisation in writing paper-worthy phylogenetic trees. Typesetting with knitr (https://cran.rstudio.com/web/packages/knitr/index.html) or Sweave (https://www.rdocumentation.org/packages/utils/versions/3.6.2/topics/Sweave) is a great way to control long and complex write-ups and style them in a way that weaves with coding input and outputs.

The principles of good writing apply to computational biology as well; unlearn bad writing habits, and learn the good ones by reading opinions or even taking courses such as Writing in the Sciences (https://www.coursera.org/learn/sciwrite) [27]. Talks and presentations in front of diverse lay and expert audiences will be a good communication exercise for you as you frame the same conversation for different audiences. As for posters, practice the general guidelines of good presentation [28]. Pay additional attention to the readability of the legend and labels. Posit images with cohesion and direction so that they can guide the eyes. Write the methodology in plain, efficient language that gets the purpose across. Australian academia also offers its wisdom in the rule of 3—your poster should be intelligible 3 metres afar, within 3 minutes of reading, and after 3 bottles of beers. Posters in the virtual conference world should be formatted to the proportions of a computer screen, and may accommodate less detail than a traditional poster, but may have advantages in the use of animations or videos to explain your science.

### Rule 10: Upon returning, reframe the narrative

As a cross-disciplinary computational researcher, you have the opportunity to reshape how these fields come together in the future. Your ideas are shaped by the theory of both fields and an understanding of the navigation rules at play in both spheres. We urge you to be bold in planning your next adventure. The data scientist who understands the difficulty and value in obtaining precious samples will think about the analysis problem accordingly. The wet lab scientist who understands the analysis constraints imposed by experimental design will plan the experiments accordingly. You, at the intersection of these 2 worlds, can imagine what is not yet possible, test your theories in silico, and plan to use your resources wisely.

Having one foot in perspectives from your home discipline, and another foot in your new discipline, is key to both being a sensible researcher and a self-compassionate one, although this is the insight that often gets forgotten. Remain focused on your hypothesis but also flexible enough to acknowledge surprising insights. Retain the confidence to question every paper you read, and the humility to accept the findings, even when they counter your beliefs. Remember that science is probationary; molecular biology more so—due to the leapfrogs in technology and biological data sciences probably most of all due to our exponential gains in genome coverage and expression data. For example, in our experience, newly sequenced genomes usurped all the dates in a well-established time tree that took months to build and edit. But the remaking the phylogeny was a good thing, as it was more complete and more accurate.

Circling back to Rule 1, remind yourself how the analysis you computed contributes to the broader field. The famous mathematician Richard Hamming once said, “When I went to lunch Friday noon, I would only discuss great thoughts after that. By great thoughts I mean ones like: ‘What will be the impact of computers on science and how can I change it?’” [29]. We would like to add that, even if you cannot answer the great question of yours, your computational biology study should have a great thought that follows it.

## Conclusions

Data science is an integral part of how we study biology now. In many ways, the modern researcher is required to be an interdisciplinary researcher, as computational workflows become part of the everyday lexicon of a project. The omics sciences are leapfrogging every day, and anyone can dabble with data, but not everyone takes that time to gain the expertise needed to form computationally or mathematically driven hypotheses. We hope that our 10 rules will prove themselves useful for those wishing to push their craft further into the interdisciplinary waters that excites us both so much.
